# Predicting the potential distribution of the Asian citrus psyllid, *Diaphorina citri* (Kuwayama), in China using the MaxEnt model

**DOI:** 10.7717/peerj.7323

**Published:** 2019-07-15

**Authors:** Rulin Wang, Hua Yang, Wei Luo, Mingtian Wang, Xingli Lu, Tingting Huang, Jinpeng Zhao, Qing Li

**Affiliations:** 1 Sichuan Agricultural University, College of Agronomy, Chengdu, Sichuan, China; 2 Sichuan Provincial Rural Economic Information Center, Chengdu, Sichuan, China; 3 Sichuan Agricultural University, Key Laboratory of Ecological Forestry Engineering of Sichuan Province/College of Forestry, Chengdu, Sichuan, China; 4 Zigong Bureau of Meteorology, Zigong, Sichuan, China; 5 Sichuan Meteorological Observatory, Chengdu, Sichuan, China; 6 Water-Saving Agriculture in Southern Hill Area Key Laboratory of Sichuan Province, Chengdu, Sichuan, China

**Keywords:** *Diaphorina citri* Kuwayama, MaxEnt, Environmental factors, Geographical distribution

## Abstract

**Background:**

Citrus huanglongbing (HLB) is a destructive disease of citrus and a major threat to the citrus industry around the world. This disease accounts for substantial economic losses in China every year. *Diaphorina citri* Kuwayama is one of the major vectors by which citrus HLB is spread under natural conditions in China. Research is needed to identify the geographic distribution of *D. citri* and its major areas of occurrence and to formulate measures for early warning, monitoring, and control of this pest and citrus HLB.

**Methods:**

In this study, the ecological niche modelling software MaxEnt (maximum entropy model) was combined with ArcGIS (a geographic information system) to predict the potential geographic distribution of *D. citri* in China. Key environmental factors and the appropriate ranges of their values were also investigated.

**Results:**

Our results show that the training data provided a good forecast (AUC_mean_ = 0.988). The highly suitable areas for *D. citri* in China are mainly concentrated to the south of the Yangtze River, and the total area is 139.83 × 10^4^ km^2^. The area of the moderately suitable areas is 27.71 × 10^4^ km^2^, with a narrower distribution than that of the highly suitable area. The important environmental factors affecting the distribution of *D. citri* were min temperature of coldest month, mean temperature of coldest quarter, precipitation of wettest quarter, mean temperature of warmest quarter, precipitation of warmest quarter, max temperature of warmest month, and temperature seasonality. These results provide a valuable theoretical basis for risk assessments and control of *D. citri*.

**Discussion:**

The predicted results showed that there were highly suitable areas for *D. citri* in Chongqing, Hubei, Anhui, and Jiangsu. Therefore, the possibility exists for the further spread of *D. citri* in China in the future. Extreme temperature variables, especially the min temperature of the coldest month, play an important role in the distribution of *D. citri* and are most closely related to the distribution of *D. citri*.

## Introduction

*Diaphorina citri* Kuwayama (Hemiptera: Liviidae) is a major pest of Rutaceae plants, including *Citrus reticulata* Blanco, *Citrus sinensis* Osbeck, *Citrus maxima* Merr., and *Murraya paniculata* Jack (Miranda et al., 2018; Yao, Zhou & Zhou, 2018). *D. citri* mainly damages the new shoots of citrus plants. Adults occur on the leaves and buds, whereas nymphs cluster on shoots, buds, and young leaves. The damaged shoots and buds wither, and the deformed leaves fall off easily, which seriously affects the growth of the plants. The white secretions from the nymphs affect photosynthesis in the branches and leaves (Yao, Zhou & Zhou, 2018). In addition to direct feeding damage, the greatest harm caused by *D. citri* is the transmission of the huanglongbing (HLB) pathogen (Manjunath et al., 2008; Fan et al., 2010). The pathogen can circulate, diffuse, and proliferate in the body of *D. citri* and has evolved a set of mechanisms to evade the immune system of the pest. When infected *D. citri* feed on healthy plants, the pathogen can be introduced into the plant through the mouthparts of *D. citri* and establish, reproduce and expand in the plant (Luo et al., 2015; Song & Luo, 2017).

Citrus HLB is a worldwide citrus disease and is the most harmful and destructive disease in the citrus industry (Luo et al., 2017). As early as the middle of the 18th century, reports were made of citrus HLB in India. At present, citrus HLB occurs in more than 40 countries in Asia, Africa, North America, and South America, which seriously threatens the development of the global citrus industry (Narouei-Khandan et al., 2016). The first report of the occurrence of citrus HLB in China was the 1920s, and the disease has spread rapidly in the southern citrus-producing areas of China. By the end of the 1970s, the disease occurred in Sichuan and Jiangxi except for Guangdong, Guangxi, Fujian, or Taiwan (Hu & Zhou, 2010). At present, 11 of the 19 provinces with citrus cultivation have been harmed by citrus HLB, and the affected area accounts for more than 80% of the total citrus cultivation area (Fan et al., 2009; Cheng et al., 2013). According to reports, citrus HLB occurred in Guangdong Province in 2016, with an area of 8 × 10^4^ hm^2^, resulting in losses of more than one billion dollars (Cheng et al., 2016).

*Diaphorina citri* was first recorded in Taiwan in 1907, and it is also believed to have originated in India (Kuwayama, 1908; Husain & Nath, 1927). Worldwide, *D. citri* is currently distributed in Asia (China, India, Sri Lanka, Malaysia, Indonesia, Philippines, Bangladesh, Thailand, Iran, Bhutan, and Afghanistan) (Bové, 2006; Sule et al., 2012; Lashkari et al., 2014; Wang et al., 2017a, 2018a), North America (USA, Mexico, Honduras, Bahamas, Cayman Islands, and Jamaica) (Halbert & Manjunath, 2004; López-Collado et al., 2013; Milosavljević et al., 2018), South America (Brazil, Colombia, Ecuador, and Paraguay) (Cornejo & Chica, 2014; Narouei-Khandan et al., 2016), Africa (Kenya, Mauritius, and Tanzania) (Shimwela et al., 2016; Rwomushana et al., 2017), and Australia (Aurambout et al., 2009). In China, *D. citri* is mainly distributed in Guangdong, Guangxi, Taiwan, Macao, Hong Kong, Fujian, Zhejiang, Jiangxi, Hunan, Guizhou, Yunnan, Sichuan, and Hainan (Wang et al., 2016).

Meteorological factors are important environmental factors affecting the distribution, occurrence, and development of pests and diseases. Global warming is a long-term increase in the average temperature of the Earth’s climate system, an aspect of climate change shown by temperature measurements and multiple effects of warming (Craufurd & Wheeler, 2009). Under the background of climate warming, the suitable areas for pests and diseases are increasing, which leads to the expansion of their geographical distributions (Monteith, 2000). Temperature is one of the main factors restricting the distribution of pests on Earth (Zhang et al., 2012). Climate warming increases the chance of insects restricted by low temperatures to spread to high altitudes (Hu et al., 2015). In recent years, with the increase of winter temperatures, the population of *D. citri* has expanded significantly, and its geographical distribution has spread northward each year, which has aggravated the speed of spread and damage scope of citrus HLB (Chen, Xu & Wang, 2016; Wang et al., 2016). Therefore, research on the influence of climate factors on the distribution of *D. citri* is important.

MaxEnt is a habitat suitability model based on the niche principle that uses species distribution data and environmental data to analyse the distribution state of species at maximum entropy (Remya, Ramachandran & Jayakumar, 2015). Compared with other niche models, the model has higher prediction accuracy and can obtain satisfactory results with relatively few distribution points (Elith et al., 2006; Petitpierre et al., 2012; Zhang et al., 2016). The MaxEnt model has been widely used by scholars in China and abroad. Kumar et al. (2014) used MaxEnt to predict the invasion potential of the exotic pest *Phenacoccus solenopsis* Tinsley (Hemiptera: Coccoidea: Pseudococcidae) in India. Penado, Rebelo & Goulson (2016) used MaxEnt to identify climatically suitable areas for bumblebees in under-sampled parts of the Iberian Peninsula. López-Martínez et al. (2016) studied the environmental suitability for *Agrilus auroguttatus* Schaeffer (Coleoptera: Buprestidae) in Mexico using MaxEnt. Lozier & Mills (2011) used the correlative niche modelling method MaxEnt to predict the geographic distribution of *Epiphyas postvittana* Walker (Lepidoptera: Tortricidae) in its native range and globally and tested model projections using known invasion data.

Recent research on *D. citri* has mainly focused on biological characteristics (Ruan et al., 2012), ecological characteristics (Onagbola et al., 2008), comprehensive control measures (Manjunath et al., 2008; Jia et al., 2015; Wang et al., 2018b), the host selection mechanism and the transmission mechanism of HLB (Inoue et al., 2009; Hijaz, Lu & Killiny, 2016; Arp, Martini & Pelz-Stelinski, 2017; Killiny et al., 2017; Yu & Killiny, 2018); research regarding prediction of its geographical distribution in China is relatively rare. To provide a theoretical basis for such prediction, risk assessments and the effective control of *D. citri*, the MaxEnt model was used to map the potential distribution of *D. citri* in China under current climate conditions, and the relationship between the distribution of *D. citri* and environmental factors was elaborated in this paper.

## Materials and Methods

### Environmental variables and species data

To obtain the occurrence records of *D. citri* in China, we accessed two online databases, the European and Mediterranean Plant Protection Organization database (EPPO, 2018) and the Global Biodiversity Information Facility database (GBIF, 2018), and consulted many published articles (Halbert & Manjunath, 2004; Yang et al., 2006; Wang et al., 2017a; Jiang et al., 2018). According to the method described by Jiang et al. (2018) to filter distribution records, we used Google Earth (Collette & Pither, 2015) to proofread the latitude and longitude. In strict accordance with the requirements of MaxEnt, duplicate records, fuzzy records and neighbouring records were removed. Finally, 135 valid records were retained for constructing the models.

In this study, to analyse the climatic suitability regionalisation of *D. citri* in China, we chose climatic factors and altitude factors as the initial environmental variables. Data on climate variables and altitude were downloaded from the official WorldClim website (Fick & Hijmans, 2017) (Table S1), which provides the average data from 1950 to 2000. MaxEnt is a mathematical model based on the principle of climate similarity; it is used to explore the correlation between geographical distribution and environmental variables (Elith et al., 2006). The choice of environmental variables is the key to determining the accuracy of the simulation. Multiple collinearity may exist among the environmental variables, which affects the model’s evaluation of response relationships and contribution rates, which in turn affects the accuracy of the simulation (Zhang & Liu, 2017). MaxEnt computes the contribution of predictor variables to model the potential occurrence of a species. In this study, we refer to Zhang & Liu’s (2017) method to screen environmental variables. The screening procedure is as follows: (1) Establishment of an initial model to calculate the contribution of the variables to the model (Table 1). (2) Use of ArcGIS to extract the attribute values for 20 variables at each of the 135 presence records and SPSS to calculate the Pearson correlation coefficients between any two variables. (3) If a correlation coefficient is greater than 0.8, the most relevant variable is retained according to the percent contribution of the variable in the initial model, and the other variable is excluded. (4) The remaining variables are sorted according to the percent contribution, and the variables with a percent contribution greater than 1.0 are retained. Through the above procedure and referring to the biological characteristics of *D. citri*, the screening of environmental variables was completed.

**Table 1:** Percent contribution and cumulative contribution of the environmental variables to the Maxent model.

Environmental variables	Code	Percent contribution	Cumulative importance
Mean temperature of coldest quarter	BIO11	33.7	33.7
Precipitation of warmest quarter	BIO18	32.6	66.3
Temperature seasonality	BIO4	23.2	89.5
Precipitation of driest month	BIO14	2.6	92.1
Precipitation seasonality	BIO15	1.6	93.7
Min temperature of coldest month	BIO6	1.3	95
Precipitation of wettest quarter	BIO16	1.2	96.2
Mean temperature of warmest quarter	BIO10	1.0	97.2
Precipitation of driest quarter	BIO17	0.6	97.8
Annual mean temperature	BIO1	0.5	98.3
Precipitation of wettest month	BIO13	0.3	98.6
Isothermality (BIO2/BIO7) (*100)	BIO3	0.3	98.9
Mean diurnal range (mean of monthly	BIO2	0.2	99.1
Precipitation of coldest quarter	BIO19	0.2	99.3
Mean temperature of wettest quarter	BIO8	0.2	99.5
Max temperature of warmest month	BIO5	0.2	99.7
Mean temperature of driest quarter	BIO9	0.1	99.8
Altitude	Alt	0.1	99.9
Temperature annual range (BIO5–BIO6)	BIO7	0.1	100
Annual precipitation	BIO12	0	100

### Modelling method and statistical analysis

MaxEnt builds a model by means of a machine-learning algorithm to predict the suitability for the occurrence of a given species in a spatial dimension. MaxEnt software (version 3.4.1), which is now open source, was downloaded from the website of the American Museum of Natural History (Phillips, Dudík & Schapire, 2019); this software has excellent predictive performance for pests and diseases (Jarnevich & Young, 2015).

The specific operational steps for MaxEnt are described herein. First, we imported the occurrence points for *D. citri* and data for 20 variables into the MaxEnt software to create the initial model. In the initial model, the ‘Random test percentage’ was set as 25, and ‘Make pictures of predictions’ and ‘Do jackknife to measure variable importance’ were chosen; the remaining model values were set to default values. Then, we evaluated the percent contributions of the environmental variables to select variables for modelling. Finally, the occurrence points and the selected environmental variables were uploaded to MaxEnt to simulate the distribution of *D. citri* in China. In the final model, ‘Random seed’ was chosen, and 10 replicate models were run. We selected the best model with the highest AUC (Area unde the receiver operating characteristic curve) value. The remaining model settings were set to the same as those in the initial model (Kumar & Stohlgren, 2009; Zhu et al., 2018).

Response curves indicate the relationship between the probability of *D. citri* occurrence and environmental variables was computed by MaxEnt. We use the response curves for each variable to avoid the influence of correlation between variables. Generally, the ecological factor value suitable for the presence of *D. citri* is generally believed to be the value when the probability of the presence of *D. citri* is greater than 0.33. ArcGIS software was used to superimpose the index distribution map with China’s administrative division map to obtain the suitability regionalization map for *D. citri*. We reclassified the distribution threshold and divided the suitable area into four categories, displaying them in different colour according to the method described by Wang et al. (2018c).

Analysis of the receiver operating characteristic (ROC) curve is an effective method for evaluating the accuracy of the species distribution model. The method uses the area under the curve (AUC) as the index to measure the model accuracy. The theoretical value range of the AUC is 0.5∼1; AUC values closer to 1 indicate a higher prediction accuracy of the model. The evaluation criteria are simulation failure (fail), 0.5 ≤ AUC < 0.6; poor simulation results (poor), 0.6 ≤ AUC < 0.7; generally fair simulation results (fair), 0.7 ≤ AUC < 0.8; good simulation results (good), 0.8 ≤ AUC < 0.9; and excellent simulation results (excellent), 0.9 ≤ AUC < 1 (Wang et al., 2007).

## Results

### Model performance

**Figure 1:**
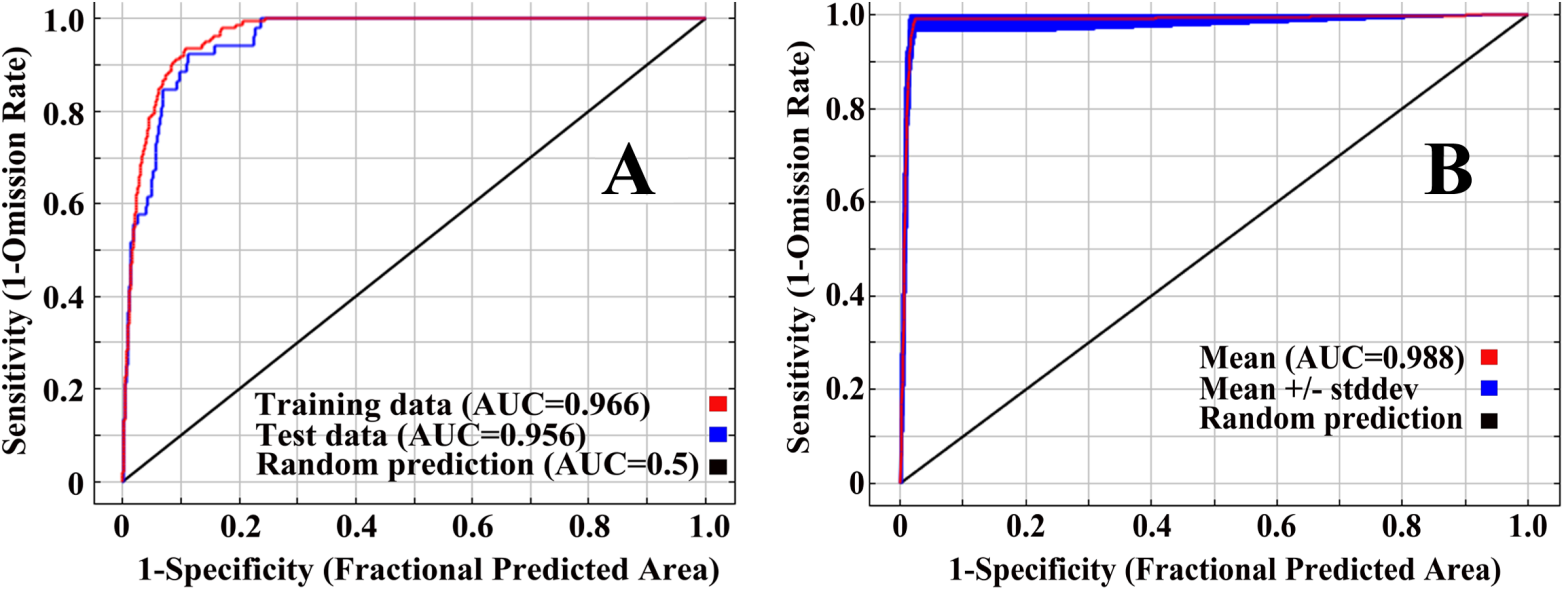
ROC curve and AUC values for the initial model (A) and the final model (B).

Figure 1A shows the ROC curve of the initial model. The AUC values of the training data and the test data are 0.966 and 0.956, respectively. According to the evaluation criteria described in ‘Materials and Methods’, the accuracy of the initial model is ‘excellent’. Figure 1B shows the ROC curve of the final model. The results show that the mean AUC value of the 10 replicates was 0.988.

### Selection of the environmental variables

Pearson correlation coefficients between the 20 environmental variables are shown in Table S2. The correlation coefficient between altitude and precipitation of warmest quarter, annual mean temperature and temperature annual range, annual mean temperature and mean temperature of wettest quarter, mean temperature of wettest quarter and precipitation of driest month, mean temperature of driest quarter and mean temperature of coldest quarter were greater than 0.8, and altitude, temperature annual range, mean temperature of wettest quarter and mean temperature of driest quarter were excluded according to the screening procedure. The results show that the percent contributions of mean temperature of coldest quarter, precipitation of warmest quarter, temperature seasonality, precipitation of driest month, precipitation seasonality, min temperature of coldest month, precipitation of wettest quarter, and mean temperature of warmest quarter were higher than 1.0%, and the cumulative sum was 97.2%, which was significantly higher than that of the other variables (Table 1). Previous biological studies have shown that extreme temperature variables play an important role in the distribution of *D. citri* (López-Collado et al., 2013). Therefore, the max temperature of warmest month was also selected for the final model. Finally, nine variables were selected: mean temperature of coldest quarter, precipitation of warmest quarter, temperature seasonality, precipitation of driest month, precipitation seasonality, min temperature of coldest month, precipitation of wettest quarter, mean temperature of warmest quarter, and max temperature of warmest month. On this basis, the final model for the distribution of *D. citri* in China was established, and the accuracy of the simulation results was evaluated.

### The potential distribution of *D. citri* in China

**Figure 2:**
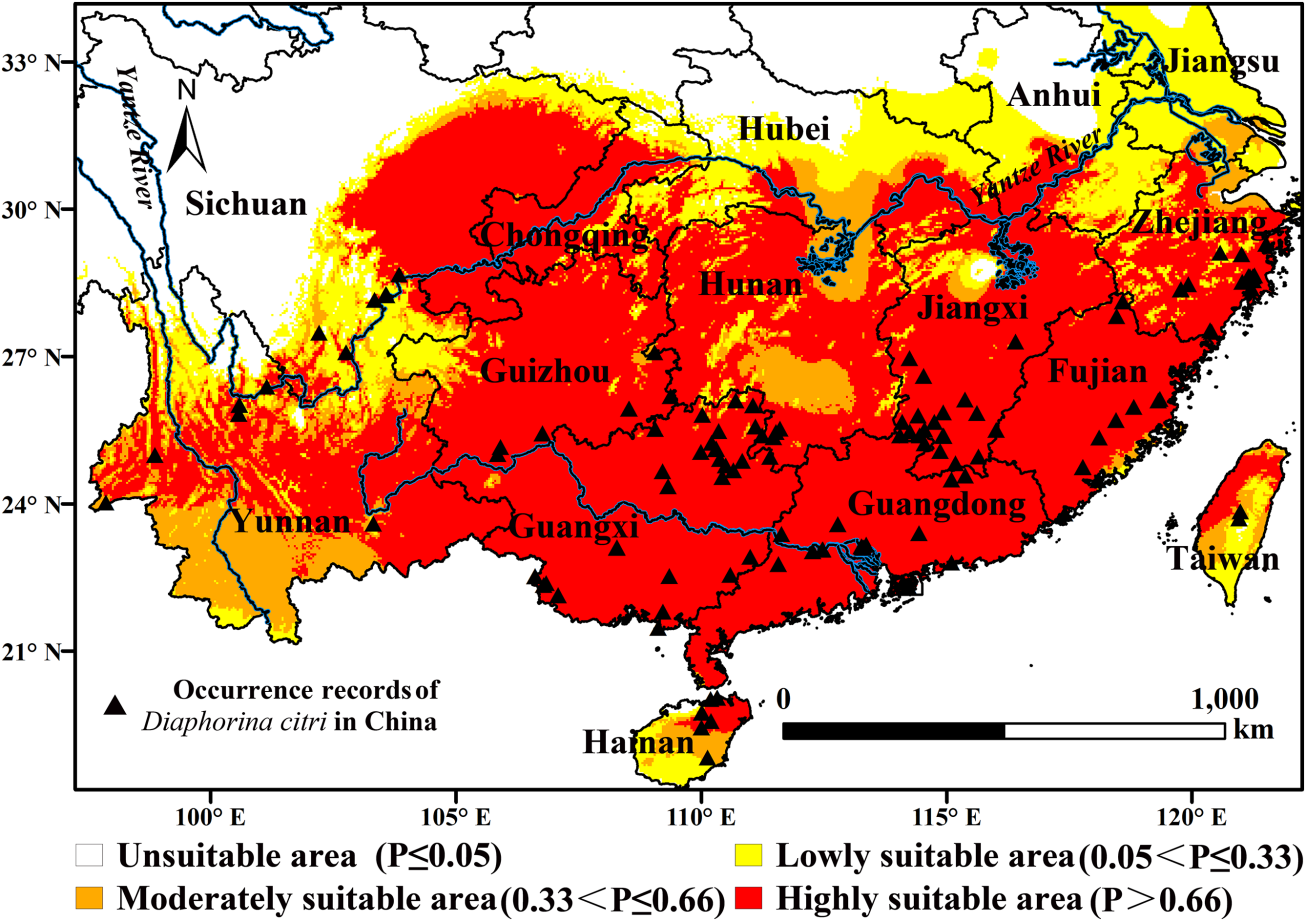
Probability (P) of the potential distribution of *D. citri* in China based on the MaxEnt model.

The selected environmental variables were combined in the MaxEnt model to obtain a suitable index distribution map of *D. citri* in China (Fig. 2). The results showed that the highly suitable areas for *D. citri* in China are mainly concentrated to the south of the Yangtze River, including Guangxi, Guangdong, Hunan, Jiangxi, Fujian, most of Guizhou, north-eastern Hainan, western Taiwan, southern, and central Zhejiang, northern Yunnan, east-central Sichuan, most of Chongqing, and Hong Kong (Fig. 2). The total area of the highly suitable area in China is 139.83 × 10^4^ km^2^, which occupies 14.52% of the area of the national territory. The moderately suitable areas are mainly distributed in southern Yunnan, southern Hubei, southern Hunan, central Hainan, southern Taiwan, southern Jiangsu, and central Zhejiang. The total area of the moderately suitable area is 27.71 × 10^4^ km^2^, with a narrower distribution than the highly suitable area. The total suitable area (the highly suitable area and the moderately suitable area) is 167.54 × 10^4^ km^2^, accounting for 17% of China’s total area.

### Key environmental variables in the model

**Figure 3:**
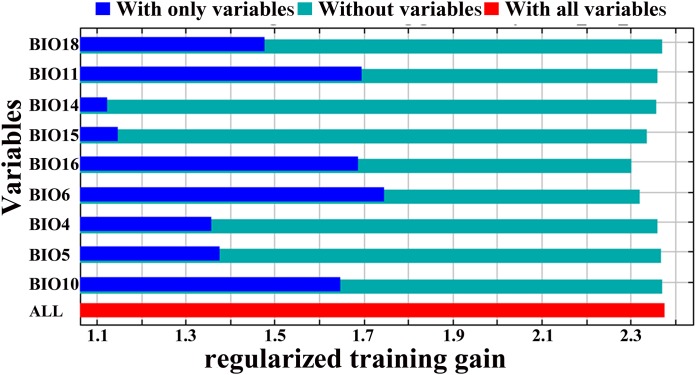
Jackknife test for variable importance in the *D. citri* suitability distribution: values shown are averages over 10 replicate runs.

The results of the jackknife test can reflect the importance of the environmental variables to the model by calculating the training gains when using ‘with only variable’, ‘without variable’, and ‘with all variables’ for the simulation. Figure 3 is the result of the analysis of the importance of environmental variables to the distribution of *D. citri* according to the jackknife test. It can be seen from the figure that the min temperature of coldest month is the most important environmental variable affecting the distribution of *D. citri* in China, and its training gain exceeds 1.7. Mean temperature of coldest quarter, precipitation of wettest quarter and mean temperature of warmest quarter were also important environmental variables, and their individual training gains were greater than 1.6. the order of importance of the nine environmental variables is min temperature of coldest month > mean temperature of coldest quarter > precipitation of wettest quarter > mean temperature of warmest quarter > precipitation of warmest quarter > max temperature of warmest month > temperature seasonality > precipitation of driest month > precipitation seasonality.

### Environmental variables affecting the occurrence of *D. citri*

**Figure 4:**
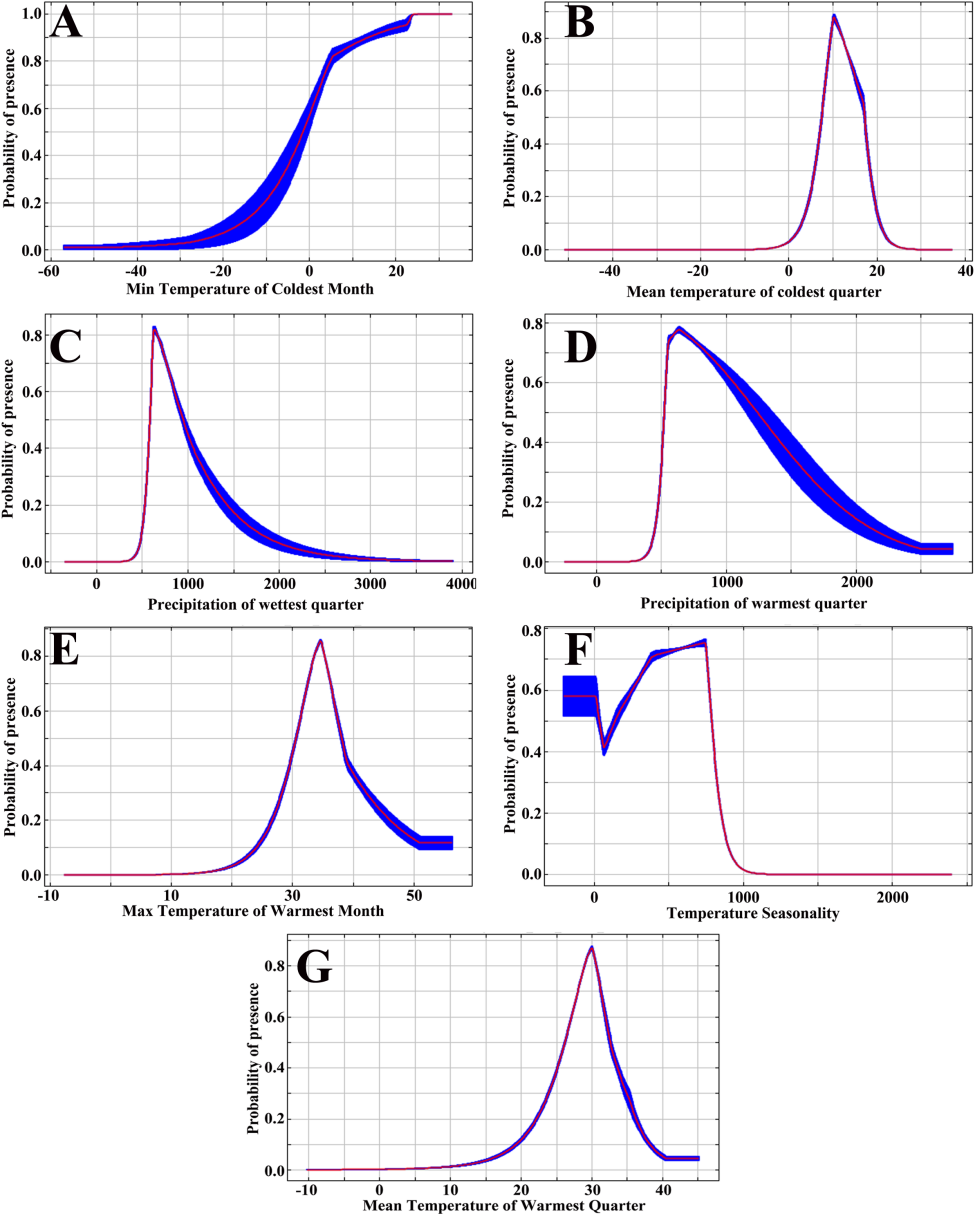
Response curves of environmental variables in the MaxEnt models: (A) BIO6; (B) BIO11; (C) BIO16; (D) BIO18; (E) BIO5; (F) BIO4; (G) BIO10.

As shown in Fig. 4, when the min temperature of coldest month is below −3.58 °C, the probability of *D. citri* presence is less than 0.33. With the increase in min temperature of coldest month, the probability increased rapidly and reached its highest point at 23.4 °C. The change in the response curve of the mean temperature of coldest quarter is different from that for the min temperature of coldest month, and the suitable range of the mean temperature of coldest quarter is 6.03–17.88 °C. When the temperature is lower than 6.03 °C or higher than 17.88 °C, the probability of *D. citri* occurrence is lower than 0.33 and reaches its highest value at 10.26 °C. When the precipitation of wettest quarter is below 548.66 mm, the probability of *D. citri* presence is lower than 0.33. With an increase in precipitation of wettest quarter, the probability of the presence of *D. citri* increased rapidly and reached its highest value at 619.02 mm. After that, the probability of *D. citri* presence slowly decreases. When precipitation of wettest quarter reaches approximately 1,189.75 mm, the probability of the presence of *D. citri* falls below 0.33. Therefore, the suitable range of precipitation of wettest quarter for *D. citri* is 562.89–1,189.75 mm.

The trends of presence probability in association with the variables (mean temperature of warmest quarter, precipitation of warmest quarter, max temperature of warmest month, temperature seasonality) are similar to that of mean temperature of coldest quarter, and the suitable ranges were 24.46–34.27 °C, 503.73–1,533.58 mm, 28.6–40.91 °C, and 56.83–818.03, respectively (Fig. 4).

## Discussion

### Evaluation of the MaxEnt model

Commonly used model evaluation indicators include overall accuracy, sensitivity, specificity, kappa, and true skill statistic (Allouche, Tsoar & Kadmon, 2006; Wang et al., 2007). The ROC curve is not affected by the threshold and is considered to be one of the best evaluation indicators at present. MaxEnt software can directly draw the ROC curve and calculate the AUC value of the model, which is convenient for judging the predictive effect of the model. Therefore, ROC curves are widely used in the evaluation of MaxEnt models. For example, Wang et al. (2017b) used ROC curves to evaluate the predictive effect of the MaxEnt model in terms of suitable habitats for the Colorado potato beetle at a global scale; Han et al. (2015) used ROC curves to determine the accuracy of niche models in predicting suitable habitats for *Bursaphelenchus xylophilus* Steiner and Bohrer (Tylenchida Thorne: Sphelenchoidae) in China. Therefore, the ROC curve is used to evaluate the predictive accuracy of the MaxEnt model. The stability of the model is verified by 10 repeated AUC values. The average AUC value of 10 replicated runs of the model was 0.988, which indicates that the simulation effect was ‘excellent’ and proves that the model can be used to simulate the potential distribution of *D. citri* in China.

### Predicting the distribution of *D. citri* in China

We used the ‘extraction’ tool in ArcGIS software to identify suitable areas for *D. citri* in China. According to previous study results, the suitable areas were divided into four grades: highly suitable areas, moderately suitable areas, low-suitability areas and unsuitable areas, and the suitable areas of each grade were calculated. The results showed that the highly suitable areas for *D. citri* in China are mainly concentrated to the south of the Yangtze River, and the total area of the highly suitable area in China is 139.83 × 10^4^ km^2^. The moderately suitable areas are distributed to the north of the highly suitable areas, and the area is 27.71 × 10^4^ km^2^. The total suitable area (the highly suitable area and the moderately suitable area) is 167.54 × 10^4^ km^2^ and accounts for 17% of China’s total area. Wang, Xiao & Zhang (2015) applied CLIMEX to predict the potential distribution of *D. citri* in China, and the results were basically consistent with our predictions, with our predicted habitat being more to the north. This difference may have occurred because of the use of different prediction models, species distribution data, and environmental variables.

The distribution of *D. citri* in China was investigated by the National Agricultural Technology Extension Service Center in 2014. The results showed that *D. citri* occurred in Zhejiang, Jiangxi, Hunan, Sichuan, Guizhou, Yunnan, Guangxi, Guangdong, and Hainan but not in Chongqing and Hubei (Wang et al., 2016). The predicted results showed that there were highly suitable areas for *D. citri* in Chongqing, Hubei, Anhui, and Jiangsu. Chongqing and Hubei are located in the core area of the citrus industry belt along the middle and upper reaches of the Yangtze River, which is one of the most suitable areas for citrus cultivation in China (Xiang, Qi & Lu, 2014). By the end of 2017, the cultivated areas of citrus in Hubei and Chongqing were 21.33 × 10^4^ and 41.28 × 10^4^ hm^2^, respectively (Ding, Yuan & Zhou, 2017). Therefore, the above two provinces had suitable conditions for the colonisation of *D. citri*. Geographically, southern Chongqing is adjacent to Sichuan, Yunnan and Hunan, while southern Hubei is adjacent to Hunan and Jiangxi. *D. citri* has been found in Sichuan, Yuan, Hunan, and Jiangxi in recent years. Since the 1980s, the occurrence boundary of *D. citri* has been moving northward (Chen, Xu & Wang, 2016). Therefore, the possibility of introduction and colonisation of *D. citri* in Chongqing and Hubei is very high, and inspection and quarantine work should be strengthened to prevent the introduction of *D. citri* to these regions. Because the climate is not suitable for citrus growth, there is almost no citrus planting in Anhui and Jiangsu, and the suitable areas for *D. citri* in Anhui and Jiangsu are very small (Wu, 2018). Therefore, we speculate that the probability of occurrence of *D. citri* in this area is very low and will not cause serious economic losses.

Hosts of *D. citri* include *Citrus reticulata* Blanco, *Citrus sinensis* Osbeck, *Citrus maxima* Merr., *M. paniculata* Jack, *M. exotica* L., and *Clausena lansium* (Lour.) Skeels. These host plants have different effects on the development, reproduction and survival of *D. citri*. Ren et al. (2018) investigated the life history of five different host plants. The results showed that the adult longevity of *D. citri* was significantly affected by the host plant and was highest on *Citrus maxima* and shortest on *Clausena lansium*, while the survivorship of larvae was highest (58.10%) on *Citrus maxima* and lowest on *Clausena lansium* (46.04%). Tsai & Liu (2000) showed that the survival rate of *D. citri* was highest on grapefruit and lowest on lime. Chen et al. (2011) measured the feeding preferences of *D. citri* among 13 citrus varieties and found that the number of *D. citri* on the shoots of Newhall navel orange and Fuju was significantly higher than that on the other 11 citrus varieties. These previous studies have shown the importance of host identity for the distribution of *D. citri*, and the analysis of the dependence of *D. citri* on hosts can help to improve the accuracy of predictions. In this paper, we did not simulate the suitable habitats of various hosts of *D. citri* in China, but we can confirm that the main citrus-producing areas cover the suitable habitats of *D. citri* according to the relevant literature. Therefore, the results of this study are still highly reliable.

### Environmental variables affecting the geographical distribution of *D. citri*

The occurrence, growth, and spread of plant diseases and insect pests depend not only on the biological characteristics of the disease and pests but also on the host plants, farming systems, management levels, and environmental conditions. Meteorological variables are extremely important environmental factors. Under other conditions that are relatively consistent, meteorological factors will become a decisive factor affecting the epidemic or large-scale outbreak of pests and diseases (Monteith, 2000; Qin et al., 2017). Studies have shown that abiotic factors such as temperature, humidity, and atmospheric pressure can affect the distribution of *D. citri* (Martini & Stelinski, 2017), and variables related to temperature are more critical in predictions (López-Collado et al., 2013). Therefore, we focus on the effect of temperature on the geographical distribution of citrus as follows.

López-Collado et al. (2013) found that the minimum temperature of the coldest month seemed to be the most important variable affecting the distribution of *D. citri*. In this paper, the importance of environmental variables was tested with the jackknife method. The results showed that the mininum temperature of the coldest month was the most important variable, indicating that it is most closely related to the distribution of *D. citri*, which is consistent with the result found by López-Collado et al. The response curve showed that the probability of the presence of *D. citri* was very low when the min temperature of coldest month was below −3.58 °C, which indicates that extremely low temperatures limit the distribution of *D. citri*. In India, Atwal, Chaudhary & Ramzan (1970) found that extremely low temperatures were not conducive to the development of the population of *D. citri*. Low temperatures below 0 °C played an important role in suppressing the population of *D. citri*, and Yang et al. (2006) found the same rule in China. Bai et al. (2008) found that the annual minimum average temperature was the main factor limiting the geographical distribution of *D. citri*. Hall, Wenninger & Hentz (2011) noted that the low temperature in winter was the main factor limiting the population growth, geographical distribution, and potential transmission of *D. citri*. These results are consistent with the results of this study.

Studies have shown that the damage caused by high temperatures to insects is irreversible and has a certain accumulative effect. Exposure to high temperatures for a long time will lead to a significant decrease in the water content of insects and even their death (Zhong, 2010; Zhang et al., 2014). Investigating the effects of high temperature on the mortality and activity behaviour of *D. citri* demonstrated that a high temperature above 40 °C leads to a decrease in the survival time of the *D. citri* population (Kuang et al., 2017). Hall, Wenninger & Hentz (2011) estimated temperature thresholds for the oviposition of *D. citri* and found that the lower and upper thresholds for oviposition were 16 and 41.6 °C, respectively. The results of this study indicate that the max temperature of warmest month is also a key variable affecting the distribution of *D. citri* and that the suitable range is 28.6–40.91 °C, which is consistent with the above conclusions. Narouei-Khandan et al. (2016) showed that the presence of *D. citri* was limited when the mean temperature in the warmest season was higher than 33 °C. The response curve of the mean temperature of warmest quarter showed that the upper limit temperature for the presence of *D. citri* was 34.27 °C. This result may have occurred because the larvae and eggs of *D. citri* are more sensitive to high temperatures than adults (Liu & Tsai, 2000). In this paper, the response curve shows the effect of a single environmental variable on species distribution, but the growth and distribution of *D. citri* depend on the comprehensive effect of various environmental factors. Therefore, this conclusion cannot fully explain the relationship between *D. citri* and the environmental variables but can be used as a theoretical reference to evaluate the relationship between them.

## Conclusions

Based on MaxEnt software and certain environmental data, this study predicts the geographical distribution of *D. citri* in China and aims to provide a scientific reference for the control of *D. citri*.

In this study, the occurrence data of *D. citri* were mainly obtained from EPPO, GBIF, and the literature, and the usable data were much fewer than the available data. The longitude and latitude of some distribution points are obtained by using positioning software, so there is inevitably some geographic error. The basic niche refers to the largest niche that is occupied by a species under the most ideal living conditions. The niche model only analyses the influence of abiotic factors on species distributions, suggesting that the niche predicted by the model is wider than the actual niche occupied by *D. citri*. Therefore, the results of this study have certain limitations and shortcomings.

Studies have shown that in the past 20 years, with increasing global warming, the growth and distribution patterns of the species have changed significantly (Zhen-Feng, Jia & Shu-Qun, 2013; Zhao et al., 2014; Pacifici et al., 2017). The lack of climate data in the past 20 years may lead to a deviation in the conclusions from the actual situation. Therefore, to ensure more reliable prediction results, more comprehensive and accurate distribution data for *D. citri* should be used, and the corresponding missing climate data should be supplemented in the next step.

## Supplemental Information

10.7717/peerj.7323/supp-1 Supplemental Information 1 List of environmental variables used for this study, with type and measurement unit.

10.7717/peerj.7323/supp-2 Supplemental Information 2 Pairwise Pearson’s correlation coefficients of environmental variables. BIO1: annual mean temperature; BIO2: mean diurnal range; BIO3: Isothermality; BIO4: temperature seasonality; BIO5: max temperature of warmest month; BIO6: min temperature of coldest month; BIO7: temperature annual range; BIO8: mean temperature of wettest quarter; BIO9: mean temperature of driest quarter; BIO10: mean temperature of warmest quarter; BIO11: mean temperature of coldest quarter; BIO12: annual precipitation; BIO13: precipitation of wettest month; BIO14: precipitation of driest month; BIO15: precipitation seasonality; BIO16: precipitation of wettest quarter; BIO17: precipitation of driest quarter; BIO18: precipitation of warmest quarter; BIO19: precipitation of coldest quarter; ALT: Altitude. The symbol ‘**’ indicates a significant correlation at the level of alpha = 0.01.

## Additional Information and Declarations

### Data Availability

The following information was supplied regarding data availability: Wang, Rulin (2019): Occurrences of *Diaphorina citri* (Kuwayama) in China. figshare. Dataset. DOI 10.6084/m9.figshare.7564685.v2.
