# Supplementary material for: Predicting the potential distribution of the Asian citrus psyllid, Diaphorina citri (Kuwayama), in China using the MaxEnt model
Source: PeerJ. 2019 Jul 15;7:e7323. doi: 10.7717/peerj.7323 (PMC6637924; doi:10.7717/peerj.7323)
Supplement: Supplemental Information 1 [file peerj-07-7323-s001.doc]

**S1 Table:**

**List of environmental variables used for this study, with type and measurement unit**

| **Code** | **Environmental variables** | **Unit** |
| --- | --- | --- |
| BIO1 | Annual Mean Temperature | ℃ |
| BIO2 | Mean Diurnal Range (Mean of monthly (max temp - min temp) | ℃ |
| BIO3 | Isothermality (BIO2/BIO7) (* 100) | - |
| BIO4 | Temperature Seasonality (standard deviation *100) | - |
| BIO5 | Max Temperature of Warmest Month | ℃ |
| BIO6 | Min Temperature of Coldest Month | ℃ |
| BIO7 | Temperature Annual Range (BIO5-BIO6) | ℃ |
| BIO8 | Mean Temperature of Wettest Quarter | ℃ |
| BIO9 | Mean Temperature of Driest Quarter | ℃ |
| BIO10 | Mean Temperature of Warmest Quarter | ℃ |
| BIO11 | Mean Temperature of Coldest Quarter | ℃ |
| BIO12 | Annual Precipitation | mm |
| BIO13 | Precipitation of Wettest Month | mm |
| BIO14 | Precipitation of Driest Month | mm |
| BIO15 | Precipitation Seasonality (Coefficient of Variation) | mm |
| BIO16 | Precipitation of Wettest Quarter | mm |
| BIO17 | Precipitation of Driest Quarter | mm |
| BIO18 | Precipitation of Warmest Quarter | mm |
| BIO19 | Precipitation of Coldest Quarter | mm |
| ALT | Altitude | m |
