# Supplementary material for: Predicting the potential distribution of the Asian citrus psyllid, Diaphorina citri (Kuwayama), in China using the MaxEnt model
Source: PeerJ. 2019 Jul 15;7:e7323. doi: 10.7717/peerj.7323 (PMC6637924; doi:10.7717/peerj.7323)
Supplement: Supplemental Information 2 — BIO1: annual mean temperature; BIO2: mean diurnal range; BIO3: Isothermality; BIO4: temperature seasonality; BIO5: max temperature of warmest month; BIO6: min temperature of coldest month; BIO7: temperature annual range; BIO8: mean temperature of wettest quarter; BIO9: mean temperature of driest quarter; BIO10: mean temperature of warmest quarter; BIO11: mean temperature of coldest quarter; BIO12: annual precipitation; BIO13: precipitation of wettest month; BIO14: precipitation of driest month; BIO15: precipitation seasonality; BIO16: precipitation of wettest quarter; BIO17: precipitation of driest quarter; BIO18: precipitation of warmest quarter; BIO19: precipitation of coldest quarter; ALT: Altitude. The symbol ‘**’ indicates a significant correlation at the level of alpha = 0.01. [file peerj-07-7323-s002.doc]

**S2 Table:**

**Pairwise Pearson’s correlation coefficients of environmental variables.**

|  | **ALT** | **BIO1** | **BIO2** | **BIO3** | **BIO4** | **BIO5** | **BIO6** | **BIO7** | **BIO8** | **BIO9** | **BIO10** | **BIO11** | **BIO12** | **BIO13** | **BIO14** | **BIO15** | **BIO16** | **BIO17** | **BIO18** |
| --- | --- | --- | --- | --- | --- | --- | --- | --- | --- | --- | --- | --- | --- | --- | --- | --- | --- | --- | --- |
| **BIO1** | 0.1 |  |  |  |  |  |  |  |  |  |  |  |  |  |  |  |  |  |  |
| **BIO2** | 0.15 | 0.37^**^ |  |  |  |  |  |  |  |  |  |  |  |  |  |  |  |  |  |
| **BIO3** | -0.05 | 0.22^*^ | 0.24^**^ |  |  |  |  |  |  |  |  |  |  |  |  |  |  |  |  |
| **BIO4** | 0.5^**^ | 0.62^**^ | 0.41^**^ | 0.13 |  |  |  |  |  |  |  |  |  |  |  |  |  |  |  |
| **BIO5** | 0.28^**^ | 0.57^**^ | 0.44^**^ | 0.13 | 0.57^**^ |  |  |  |  |  |  |  |  |  |  |  |  |  |  |
| **BIO6** | -0.11 | 0.44^**^ | 0.15 | 0.06 | 0.4^**^ | 0.5^**^ |  |  |  |  |  |  |  |  |  |  |  |  |  |
| **BIO7** | 0.25^**^ | 0.81^**^ | 0.36^**^ | 0.14 | 0.68^**^ | 0.61^**^ | 0.46^**^ |  |  |  |  |  |  |  |  |  |  |  |  |
| **BIO8** | 0.15 | 0.91^**^ | 0.22^*^ | -0.08 | 0.49^**^ | 0.64^**^ | 0.41^**^ | 0.71^**^ |  |  |  |  |  |  |  |  |  |  |  |
| **BIO9** | -0.03 | 0.62^**^ | 0.21^*^ | 0.02 | 0.59^**^ | 0.41^**^ | 0.72^**^ | 0.58^**^ | 0.55^**^ |  |  |  |  |  |  |  |  |  |  |
| **BIO10** | 0.3^**^ | 0.59^**^ | 0.53^**^ | 0.16 | 0.69^**^ | 0.52^**^ | 0.22^**^ | 0.58^**^ | 0.53^**^ | 0.46^**^ |  |  |  |  |  |  |  |  |  |
| **BIO11** | -0.07 | 0.56^**^ | 0.2^*^ | 0.01 | 0.49^**^ | 0.36^**^ | 0.75^**^ | 0.55^**^ | 0.54^**^ | 0.8^**^ | 0.44^**^ |  |  |  |  |  |  |  |  |
| **BIO12** | 0.33^**^ | 0.53^**^ | 0.37^**^ | 0.13 | 0.77^**^ | 0.48^**^ | 0.24^**^ | 0.59^**^ | 0.6^**^ | 0.49^**^ | 0.59^**^ | 0.33^**^ |  |  |  |  |  |  |  |
| **BIO13** | 0.13 | 0.3^**^ | 0.17^*^ | -0.07 | 0.59^**^ | 0.3^**^ | 0.39^**^ | 0.29^**^ | 0.29^**^ | 0.49^**^ | 0.41^**^ | 0.57^**^ | 0.38^**^ |  |  |  |  |  |  |
| **BIO14** | -0.11 | 0.78^**^ | 0.31^**^ | 0.05 | 0.36^**^ | 0.53^**^ | 0.55^**^ | 0.7^**^ | 0.81^**^ | 0.72^**^ | 0.48^**^ | 0.59^**^ | 0.37^**^ | 0.12 |  |  |  |  |  |
| **BIO15** | -0.01 | 0.1 | 0.73^**^ | 0.14 | 0.19^*^ | 0.31^**^ | 0.14 | 0.06 | 0.05 | 0.09 | 0.39^**^ | 0.07 | 0.21^*^ | 0.14 | 0.18^*^ |  |  |  |  |
| **BIO16** | 0.71^**^ | 0.28^**^ | 0.17^*^ | -0.08 | 0.7^**^ | 0.39^**^ | 0.05 | 0.43^**^ | 0.44^**^ | 0.23^*^ | 0.45^**^ | 0.1 | 0.51^**^ | 0.33^**^ | 0.1 | 0.02 |  |  |  |
| **BIO17** | 0.38^**^ | 0.61^**^ | 0.3^**^ | 0.01 | 0.72^**^ | 0.57^**^ | 0.6^**^ | 0.68^**^ | 0.6^**^ | 0.79^**^ | 0.59^**^ | 0.62^**^ | 0.62^**^ | 0.4^**^ | 0.69^**^ | 0.14 | 0.55^**^ |  |  |
| **BIO18** | **0.92^**^** | 0.22^*^ | 0.23^**^ | -0.01 | 0.62^**^ | 0.39^**^ | 0.02 | 0.35^**^ | 0.31^**^ | 0.11 | 0.42^**^ | 0.08 | 0.42^**^ | 0.27^**^ | 0 | 0.07 | 0.74^**^ | 0.5^**^ |  |
| **BIO19** | 0.33^**^ | 0.66^**^ | 0.32^**^ | 0.03 | 0.75^**^ | 0.56^**^ | 0.49^**^ | 0.73^**^ | 0.59^**^ | 0.71^**^ | 0.6^**^ | 0.69^**^ | 0.63^**^ | 0.4^**^ | 0.66^**^ | 0.1 | 0.51^**^ | 0.79^**^ | .44^**^ |

**Notes:**

BIO1: Annual Mean Temperature; BIO2: Mean Diurnal Range; BIO3: Isothermality; BIO4: Temperature Seasonality; BIO5: Max Temperature of Warmest Month; BIO6: Min Temperature of Coldest Month; BIO7: Temperature Annual Range; BIO8: Mean Temperature of Wettest Quarter; BIO9: Mean Temperature of Driest Quarter; BIO10: Mean Temperature of Warmest Quarter; BIO11: Mean Temperature of Coldest Quarter; BIO12: Annual Precipitation; BIO13: Precipitation of Wettest Month; BIO14: Precipitation of Driest Month; BIO15: Precipitation Seasonality; BIO16: Precipitation of Wettest Quarter; BIO17: Precipitation of Driest Quarter; BIO18: Precipitation of Warmest Quarter; BIO19: Precipitation of Coldest Quarter; ALT: Altitude. The symbol ‘**’ indicates a significant correlation at the level of alpha = 0.01.
